# A Study on the Process, Microstructure, and Properties of Laser Additive Manufacturing of Titanium Alloys

**DOI:** 10.3390/ma19102104

**Published:** 2026-05-16

**Authors:** Xuqiang Kang, Bingqi Wang, Anguo Huang

**Affiliations:** School of Materials Science and Engineering, Huazhong University of Science and Technology, Wuhan 430074, China

**Keywords:** tissue properties, section scanning, laser additive manufacturing

## Abstract

To eliminate lack-of-fusion (LoF) defects at partition interfaces during laser powder bed fusion of titanium alloys, this study proposes a multi-layer, partitioned-scanning strategy with staggered interfaces. The strategy was optimized through an orthogonal experimental design focused on single-track morphology. Experimental results indicate that this approach effectively suppresses defects in quadrant-partitioned samples, significantly improving the relative density from 93.6% to 97.9%. In addition, the strategy markedly refines the microstructure and enhances microhardness. Mechanical testing confirms that the fabricated components achieve performance comparable to those produced by conventional continuous scanning.

## 1. Introduction

Titanium alloys are characterized by low density, high specific strength, and excellent high-temperature mechanical and corrosion resistance properties, making them widely used in critical structural components such as aerospace main spars, stringers, and fasteners [1,2,3]. The demand for lightweight aerospace systems has led to the widespread adoption of large-scale monolithic titanium alloy structures. However, conventional manufacturing routes that combine forging and extensive machining are characterized by prolonged lead times, significant material waste, and unfavorably high buy-to-fly ratios, which together impede the rapid development of defense equipment [4,5,6].

Additive manufacturing (AM), an emerging technology that transcends traditional manufacturing constraints, integrates mechanical automation, materials science, and computer-aided design. It enables the near-net-shape fabrication of diverse materials and intricate structural components, offering superior design flexibility and exceptional versatility [7,8,9]. Specifically, laser metal deposition (LMD) is a prominent branch of AM and serves as a primary process for the rapid prototyping of metallic structural components. Due to its high deposition efficiency, LMD significantly reduces manufacturing lead times, making it a highly promising technology for fabricating large-scale titanium-alloy components [10,11,12,13].

Currently, zone-scanning strategies are commonly employed in the laser metal deposition (LMD) of large-scale titanium alloy components to enhance fabrication quality [14,15]. However, these strategies often increase the risk of defect formation, particularly voids and inter-zonal lack of fusion [16,17]. Valente et al. [18] reported that zone scanning induces significant lack-of-fusion defects between “island” regions. These defects are irregularly shaped and distributed periodically along the boundaries. Furthermore, Lu et al. [19] confirmed that checkerboard scanning often leads to localized uneven heat accumulation due to complex boundary overlap. In their study of TC11 titanium alloy, this phenomenon led to microporosity, reducing the relative density to approximately 98.67%. Consequently, as highlighted by Khorasani et al. [20], optimized scanning path planning is essential for controlling the density of complex components.

Furthermore, partitioned scanning strategies modify the local thermal cycling of the component, significantly influencing its microstructure and mechanical properties [21].

Furthermore, partitioned scanning strategies modify the local thermal cycling of the component, significantly influencing its microstructure and mechanical properties [21]. Gushchina et al. [22] reported that different scanning strategies in laser direct energy deposition alter the thermal history of Ti-6Al-4V, thereby affecting phase transformation and mechanical properties. Zheng et al. [23] found that scanning strategies in selective laser-melted Ti-6Al-4V significantly affect surface morphology, microstructure, microhardness, and quasi-static and dynamic mechanical properties, with the 0° scanning strategy showing the most pronounced anisotropy. Dejene et al. [24] indicated that these strategies shape the local thermal history of the deposit, which may lead to grain coarsening and microstructural inhomogeneity. Yang et al. [25] demonstrated that an intermittent island-based scanning strategy effectively reduces grain size, thermal accumulation, and anisotropic residual stresses, outperforming traditional strip-based or continuous methods. However, they also noted that island- and island-strip-scanning tend to generate more lack-of-fusion defects, thereby increasing overall porosity. This risk is particularly pronounced during the fabrication of large-scale components. Zhang et al. [26] showed that refining checkerboard partitioning and varying the interlayer scanning path can effectively mitigate heat accumulation and disrupt directional solidification in Ti6Al4V alloys.

Effectively suppressing defects and enhancing microstructural and mechanical properties remain critical challenges when formulating partitioned scanning strategies. This study employs an orthogonal experimental design to determine the optimal process parameters for single-pass laser metal deposition (LMD) of titanium alloys [27,28]. Based on these parameters, a scanning strategy featuring interlayer-staggered partitioning is proposed. By staggering the partition boundaries between layers, this strategy effectively mitigates the formation of defects such as a lack of fusion. Experimental results on component-scale parts demonstrate that this interlayer-staggered zoning approach yields superior overall microstructure and mechanical performance compared to conventional strategies. In fact, local metrics approach the levels achieved through continuous scanning. Ultimately, this study offers new insights into controlling the quality and properties of large-scale titanium alloy components during laser metal deposition.

## 2. Materials and Methods

### 2.1. Experimental Materials and Equipment

**Figure 1:**
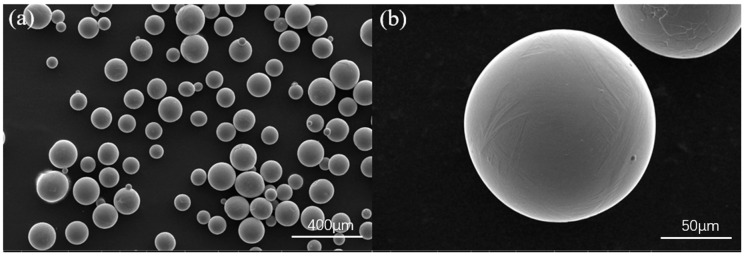
SEM morphologies of PREP TC4 powder. (**a**) low-magnification overview (100×); (**b**) high-magnification surface details (800×).

The TC4 titanium alloy powder used in this study was produced via the plasma rotating electrode process, with particle sizes ranging from 53 to 150 μm. Its chemical composition and initial microstructure are presented in Table 1 and Figure 1, respectively. Before the experiments, the powder was dried in an electric oven at 120 °C for 2 h. Annealed and rolled TC4 plates were selected as substrates. To ensure strong metallurgical bonding between the substrate and the deposited layer, the substrate surfaces were first degreased with acetone. Subsequently, the plates were chemically cleaned using an acid solution (HF:HNO_3_:H_2_O = 1:3:30 by volume) to remove the surface oxide layer.

The laser deposition system utilized in this study primarily comprises a laser and optical delivery system, a powder feeder, a motion control unit, and systems for inert gas shielding, water cooling, and centralized control. Specifically, the setup employs an IPG YLS-4000 fiber laser (IPG Photonics GmbH & Co., KG, Burbach, Germany) and a DPSF-2 dual-chamber powder feeder (Beijing Aeronautical Manufacturing Technology Research Institute, Beijing, China). During the deposition process, the substrate remains stationary while a KUKA-KR60HC six-axis robotic arm (KUKA SE & Co., KGaA, Augsburg, Germany) manipulates the laser processing head and the coaxial powder nozzle to achieve precise material deposition. The laser focal plane was located on the substrate surface, corresponding to a defocus distance of 0 mm. The laser spot diameter on the deposition surface was approximately 3 mm.

### 2.2. Testing Methods

In this experiment, a DK7745 wire electrical discharge machining (WEDM) tool (Taizhou, Jiangsu, China) was employed to extract cross-sectional specimens from the laser-deposited samples. These sections, encompassing the deposited zone, heat-affected zone (HAZ), and substrate, were sequentially ground using SiC abrasive papers (200, 400, 800, and 1200 grit) and polished with a 0.05 μm alumina suspension. To reveal the microstructure, the specimens were etched using Kroll’s reagent (2 mL HF, 6 mL HNO_3_, and 92 mL H_2_O). Finally, the cross-sectional morphology of the deposited tracks was observed and characterized using a stereo microscope (Stemi 508, Carl Zeiss Microscopy GmbH, Jena, Germany).

The relative density of the specimens, fabricated via continuous and partitioned scanning strategies, was determined using Archimedes’ principle. An electronic balance was used to measure the weight of each specimen in both air and distilled water. The density of the specimen was then calculated from the weight difference between the two media. Finally, the relative density was determined as the ratio of the measured density to the theoretical density of the TC4 alloy. The formula for calculating relative density is expressed as follows:

(1) $$\rho^{\prime }=\frac{G_{air}\times \rho_{H_{2}O}}{G_{air}-G_{H_{2}O}}/\rho_{o}$$

In the equation, represents the weight of the test specimen in air, represents the weight of the test specimen in water, represents the density of water, and represents the theoretical density of TC4 titanium alloy, which is taken here as 4.42 g/cm^3^. The test specimens used in the experiment are those intended for tensile property testing.

**Figure 2:**
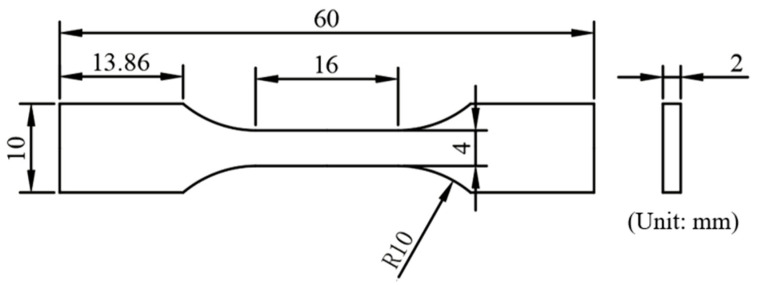
Dimensions of the tensile specimen.

In this study, tensile tests were performed on specimens fabricated using the optimized zone-scanning strategy, following the GB/T 228.1-2021 standard [29]. The specimens were machined to the dimensions illustrated in Figure 1 and Figure 2, and their surfaces were ground with SiC abrasive papers to ensure a consistent finish. Tensile testing was conducted at room temperature using a Shimadzu AG-IC universal testing machine (Shimadzu Corporation, Kyoto, Japan) at a crosshead speed of 1 mm/min, during which the tensile strength, yield strength, and elongation were recorded. To investigate the influence of scanning strategies on the tensile properties, fracture morphologies were characterized using a field-emission scanning electron microscope (Quanta 650 FEG, FEI Company, Hillsboro, OR, USA). Additionally, the microhardness of the optimized specimens was measured using a 430SVD Vickers tester (Wilson Hardness, Buehler Ltd., Lake Bluff, IL, USA) with a 9.8 N (1 kgf) load and a 15 s dwell time.

### 2.3. Optimization of Process Parameters for Single-Pass Scanning

Laser power, scanning speed, and powder feed rate are the primary parameters in the laser metal deposition (LMD) process. The powder feed rate refers to the mass of powder delivered by the powder feeding system per unit time, rather than the velocity of individual powder particles. Therefore, it is expressed as a mass flow rate with the unit of g/min. Following the additive manufacturing principle, in which structural integrity is built from individual tracks and layers, the final quality of a component is fundamentally determined by the quality of each deposition pass. Consequently, a three-factor, four-level orthogonal experiment was conducted using an L16 orthogonal array (Table 2). The single-pass deposition length was set to 50 mm to determine the optimal process parameters for subsequent investigations.

### 2.4. Design of Partition Scanning Strategies

**Figure 3:**
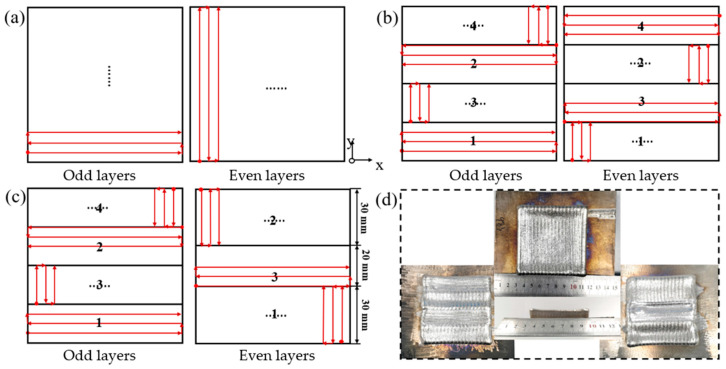
Scanning strategies for the fabrication of block components and the physical specimen. (**a**) Continuous scanning strategy. (**b**) Zone 1 scanning strategy. (**c**) Zone 2 scanning strategy. (**d**) Physical specimen.

Two strategies were employed to fabricate block structures (80 × 80 × 15 mm^3^): a continuous scanning strategy with orthogonal inter-layer vectors, and a partitioned strategy featuring sequential jumps between four rectangular sub-regions without inter-zonal gaps (hereafter denoted as “Sub-region 1”). For the Sub-region 1 strategy, the y-direction was divided into four equal segments (80 × 20 mm^2^ each). The scanning vectors were mutually perpendicular across both consecutive layers and adjacent sub-regions within the same layer. The detailed scanning paths for the odd and even layers are illustrated in Figure 3a,b.

To mitigate porosity and lack-of-fusion defects at the sub-region boundaries—often caused by incomplete powder melting in the Sub-region 1 strategy—a scanning strategy with variable interlayer partition sizes is proposed. Specifically, the partition dimensions for odd and even layers differ, effectively offsetting the interlayer boundaries. This approach utilizes the laser heat from the subsequent layer to remelt powder at the prior boundaries, thereby reducing localized porosity. This layer-wise variable strategy (hereafter abbreviated as “Partition 2”) is illustrated in Figure 3c. While the scanning pattern for odd layers remains identical to Sub-region 1, the even layers are divided into three sub-regions: two outer regions measuring 80 × 30 mm^2^ and a central region measuring 80 × 20 mm^2^. The scanning vectors are maintained perpendicular between adjacent sub-regions within a layer and across consecutive layers.

## 3. Results

### 3.1. Determination of the Optimal Process Parameters

**Figure 4:**
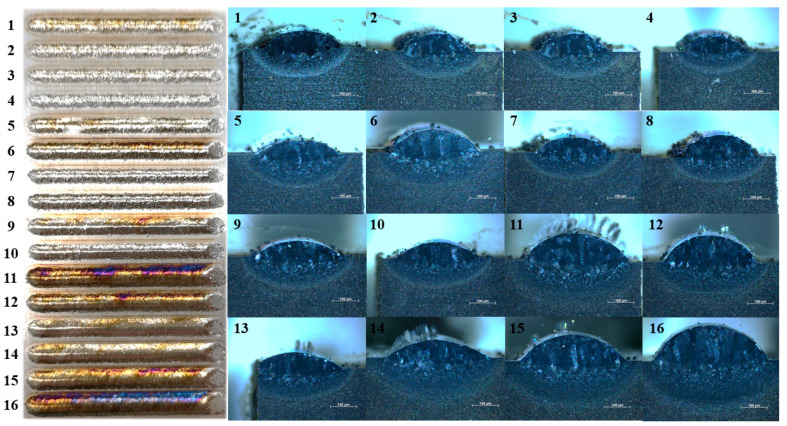
Surface and cross-sectional morphologies of a single-pass cladding track. The scale bar is 100 μm.

To determine the optimal laser metal deposition parameters, single-pass experiments were conducted according to the orthogonal array presented in Table 2. The resulting surface and cross-sectional morphologies of the deposited tracks are illustrated in Figure 4, where each label corresponds to the trial number in the orthogonal array.

As illustrated in the figure, no obvious internal defects are observed within the deposited tracks. However, at a laser power of 1100 W, the powder particles failed to melt completely due to the insufficient energy input, leading to severe powder adhesion on the surfaces of tracks 1–4. Similar adhesion occurred on tracks 7 and 8, which was attributed to the excessively high powder feed rate. For tracks 11 and 16, a mismatch between laser power and scanning speed caused excessive heat accumulation, resulting in severe track oxidation. In addition to bead morphology and internal defects, dilution is also an important indicator for evaluating the quality of single-track deposits. In this study, dilution was qualitatively evaluated from the cross-sectional morphologies in Figure 4, mainly according to the penetration depth and the melted substrate region below the original substrate surface. For samples 1–4, 7, 8, and 10, the relatively low heat input resulted in insufficient melting of the substrate and powder, leading to a shallow penetration region and low dilution. This weak metallurgical bonding may increase the risk of lack-of-fusion defects. In contrast, samples 11 and 16 exhibited excessive heat input due to the mismatch between laser power and scanning speed, resulting in a deeper melted substrate region and excessive dilution, accompanied by severe oxidation during deposition. Therefore, neither insufficient nor excessive dilution is favorable for obtaining stable deposited beads. Samples 12 and 15 showed relatively smooth bead surfaces, fewer internal defects, and moderate penetration into the substrate, indicating a more appropriate dilution level and better metallurgical bonding. To ensure high efficiency for the subsequent fabrication of bulk structures, the geometries of the remaining tracks were evaluated. As shown in Table 3, the heights of tracks 12 and 15 both reached 1 mm. To maintain a high deposition rate relative to the track width as well, the parameters for track 15 were ultimately selected as the optimal settings. These specific parameters include a laser power of 2000 W, a scanning speed of 8 mm/s, and a powder feed rate of 4.44 g/min.

### 3.2. Density and Fabrication Defects in Titanium Alloy Components Produced via Laser Metal Deposition

The block component was fabricated using the optimal process parameters established in Section 3.1. Figure 3d presents a photograph of the as-deposited part. As shown, all three scanning strategies yielded satisfactory macro-morphology and structural integrity. The component produced via the continuous scanning strategy exhibits a smoother surface finish. In contrast, those fabricated using zone scanning strategies display visible overlaps on the surface, which leads to reduced flatness for both the top and lateral surfaces.

**Figure 5:**
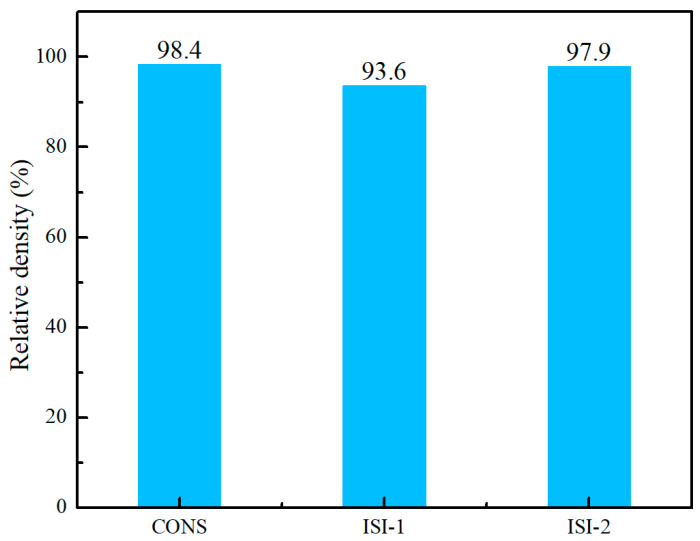
Comparison of relative density for specimens produced by continuous and two partitioned scanning strategies.

Relative density is a critical metric governing the mechanical performance of fabricated components. In this study, tests were performed via Archimedes’ principle to evaluate the impact of various scanning strategies on component density. For each strategy, three tensile specimens oriented in the y-direction were selected to measure relative density, with the mean values presented in Figure 5. As illustrated, the Zone 1 (ISI-1) strategy yielded the lowest relative density at approximately 93.6%. Conversely, the continuous scanning strategy attained the highest density of 98.4%, followed closely by Zone 2 (ISI-2) at 97.9%, which showed no significant deviation from the continuous strategy.

**Figure 6:**
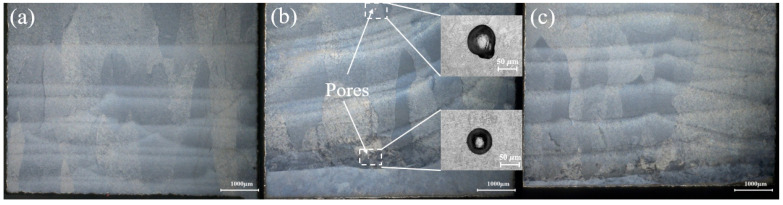
Comparison of the cross-sectional morphologies of the specimens. (**a**) Continuous scanning strategy. (**b**) Zone 1 scanning strategy. (**c**) Zone 2 scanning strategy.

To further investigate the impact of zone-scanning strategies on component density, specimens were cross-sectioned at the sub-region boundaries near the substrate for detailed observation. Figure 6 compares the cross-sectional morphologies obtained via digital microscopy. The results show that the specimens from the continuous and Zone 2 scanning strategies exhibit high fusion integrity with no discernible voids. In contrast, the Zone 1 specimen contains a high density of microscopic pores, with diameters averaging approximately 50 μm. This confirms that the proposed interlayer variable-dimension zoning strategy effectively mitigates defects, such as porosity, at inter-zonal boundaries.

Notably, the prescribed overlap rate of 50% provided sufficient overlap between regions, precluding lack-of-fusion (LOF) defects caused by insufficient overlap dimensions. This suggests that the primary cause of LOF voids is the overlapping scheme, particularly when scanning vectors are perpendicular to those of adjacent sub-regions. In continuous scanning, each layer is scanned in a uniform direction without partitioning, resulting in relatively smooth track-to-track overlap morphology. In contrast, under the Partition 1 strategy, the zig-zag scanning pattern causes powder to accumulate at sub-region boundaries during the turnaround points of the laser path. Consequently, during the deposition of the subsequent layer, the localized powder buildup prevents full penetration by the laser energy, leading to LOF voids. The Zone 2 strategy addresses this by offsetting sub-region boundaries across layers. As the heat source crosses these previous boundary coordinates at a constant velocity in the next layer, localized powder stagnation is eliminated. Any unmelted powder from the previous layer is effectively remelted by the high-energy density of the current melt pool, significantly reducing boundary-edge defects.

### 3.3. Microstructural Analysis

**Figure 7:**
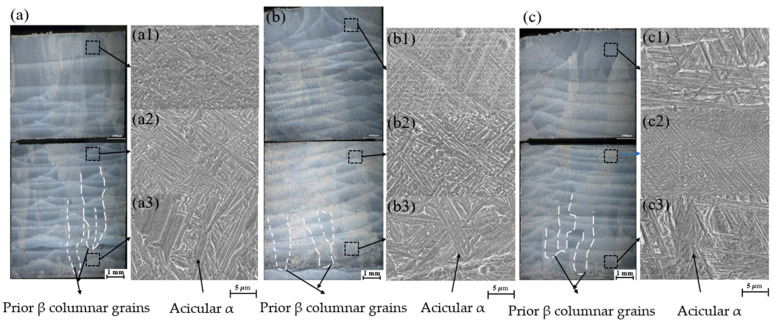
Surface morphologies and microstructures of the fabricated specimens under different scanning strategies. (**a**) Continuous scanning strategy. (**b**) Zone 1 scanning strategy. (**c**) Zone 2 scanning strategy. For each scanning strategy, the images labeled 1, 2, and 3 correspond to the top, middle, and bottom regions of the formed part, respectively.

The microstructure of the fabricated component is a critical factor that determines its performance. We utilized a deep-field 3D microscope and a scanning electron microscope to examine the cross-sectional morphology across three scanning strategies. The analysis included the microstructure at various locations, including the region near the substrate, the middle section, and the area near the surface, as illustrated in Figure 7. Figure 7a–c shows that coarse primary β-columnar grains formed under all scanning strategies and spanned multiple deposition layers. This observation suggests that the growth of β grains parallel to the deposition direction may be related to directional heat dissipation through the substrate. These columnar grains are finer at the bottom of the workpiece and become coarser toward the top. This trend may be attributed to the increased heat accumulation and reduced heat dissipation as the deposition height increases [30]. During the laser metal deposition process, heat accumulation intensifies as the number of deposited layers increases. Consequently, the cooling and solidification rates at the top are expected to be lower than those at the bottom, which may promote the formation of coarser columnar grains. However, the dimensions of the β-columnar grains near the substrate vary depending on the scanning strategy. Under the continuous scanning strategy, the width of the β-columnar grains ranges from 350 μm to 1200 μm. For the Zone 1 scanning strategy, the width ranges from 300 μm to 1000 μm. Under the Zone 2 scanning strategy, the width ranges from 300 μm to 800 μm, which is smaller than the widths observed under the other two strategies. This difference may be associated with repeated reheating of previously deposited tracks during continuous scanning, which can promote further coarsening of β grains. Furthermore, the cyclic heating of preceding layers produces banded structures in the cross-section of the component. These structures create a micro-heat-affected zone that appears dark under an optical microscope after etching. This phenomenon makes the boundaries of each deposition layer clearly visible.

Furthermore, as shown in Figure 7(a1–c3), there are significant differences in the microstructure among the bottom, middle, and top of the formed part. These differences occur primarily because the cooling rates vary across different regions. When the molten pool cools from the high-temperature zone to the two-phase zone, various α + β microstructures are produced [31]. At the bottom of the formed part, the first few layers undergo high cooling rates due to direct contact with the substrate. This process results in an acicular α’ morphology, which is visible in Figure 7(a3,b3,c3). During solidification, the maximum temperature gradient drives these acicular α’ phases to grow into elongated shapes that are perpendicular to the solid–liquid interface. As the number of deposited layers increases, the accumulated heat within the part rises, and the cooling rate continues to decrease. This transition leads to the preferential formation of a lamellar α morphology that exhibits a basket-weave microstructure, as shown in Figure 7(a2,b2,c2). At the top of the deposited layers, significant thermal diffusion causes disorder in certain directions. This phenomenon results in the formation of shorter lamellar α phases and a finer basket-weave microstructure, as shown in Figure 7(a1,b1). Under the Zone 2 scanning strategy, some acicular α’ martensite even formed at the top of the part, as shown in Figure 7(c1). This observation indicates that the cooling rate at the top under the Zone 2 scanning strategy is lower than the rates under the continuous and Zone 1 scanning strategies.

An examination of the microstructure in Figure 7 reveals that the thickness of the acicular α’ phases is smaller under the two partitioned scanning strategies than under the continuous scanning strategy. Additionally, the aspect ratio of the lamellar α phases is lower than the ratio observed in parts formed under the continuous scanning strategy. These results indicate that the α lamellae are more refined under the partitioned scanning strategies. The analysis suggests that the partitioned scanning method reduces continuous heat accumulation in previously deposited tracks. This reduction occurs because the method shortens the scan line length and introduces jumps between subregions. This approach facilitates heat diffusion and prevents severe thermal accumulation. As the heat source moves away, the previously deposited channels experience increased undercooling. This phenomenon increases the nucleation rate of α phases in different orientations within the tracks, leading to smaller grain sizes. Compared to the Zone 1 scanning strategy, the Zone 2 strategy results in a smaller thickness of α lamellae in the central region. Furthermore, some acicular α’ phases precipitate at the top under the Zone 2 strategy. This difference arises because the short-side scanning length of the even-numbered layers in Zone 2 is 30 mm, which is longer than the 20 mm used in Zone 1. Zigzag scanning with a shorter scan length dissipates heat more slowly, making it easier for heat to accumulate [32]. Therefore, the rational selection of the scanning vector length for each zone is essential.

### 3.4. Microhardness

**Figure 8:**
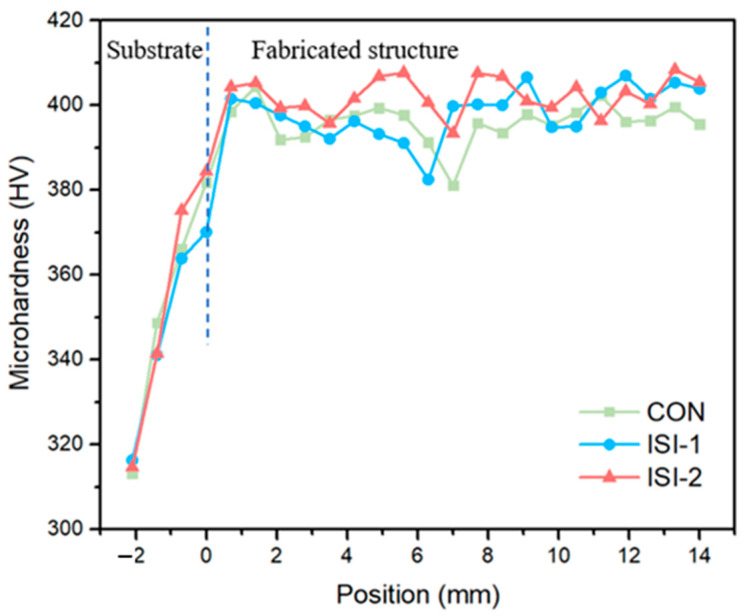
Vertical microhardness profiles of the cladded layers under different scanning strategies.

Microhardness was measured on the deposited layers at various heights along the cross-sections for each scanning strategy. These measurements were performed using a Vickers hardness tester. The spacing between measurement points along the height direction was 700 μm. A total of 24 sets of points were measured. At each height, three hardness values were obtained at intervals of 500 μm. The average of these three values was defined as the hardness of the deposited layer at that specific height. Figure 8 shows the results.

The microhardness results indicate that the hardness of the fabricated components exceeds that of the TC4 substrate across all scanning strategies. Furthermore, fluctuations in the hardness distribution are observed at different heights within the deposited layers. These local variations may be related to the repeated thermal cycling during layer-by-layer deposition, in which subsequent layers reheat or partially remelt the previously solidified regions, thereby affecting the local microstructure and hardness distribution. As shown in the figure, the deposited layers near the substrate exhibit relatively higher hardness under all scanning strategies, which may be associated with the formation of acicular α′ martensite in these regions. In addition, differences in hardness can be observed among the three scanning strategies. The deposited component fabricated using the Zone 2 scanning strategy tends to show higher microhardness than those fabricated using the continuous and Zone 1 scanning strategies. This may be attributed to the finer microstructure formed under the Zone 2 scanning strategy, as discussed above. The smaller grain size and higher density of grain boundaries can more effectively hinder dislocation motion and increase the resistance to plastic deformation, resulting in relatively higher hardness.

### 3.5. Mechanical Properties

**Figure 9:**
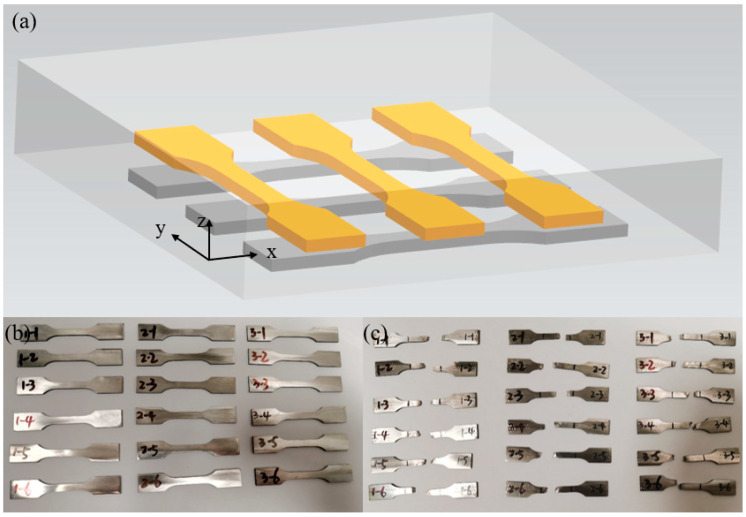
Schematic of the tensile specimen extraction locations and photographs of the specimens. (**a**) Schematic of the extraction locations. (**b**) Physical specimens before testing. (**c**) Fractured specimens.

Because the zone scanning strategy involves dividing the workpiece into multiple sub-zones, it is prone to introducing lack-of-fusion defects at the boundaries between these zones. These defects can subsequently affect the mechanical properties of the fabricated component. In this study, we selected three tensile specimens in each of two specific directions at the same horizontal position. These directions include the intra-zone direction along the x-axis and the inter-zone direction along the y-axis. As illustrated in Figure 9a, the black specimens represent the x-direction and the yellow specimens represent the y-direction. We extracted these specimens according to the dimensions specified in Section 3.4 to investigate how different scanning strategies influence the tensile properties in both directions. Figure 9b displays the physical specimens, which have an original gauge length of 12 mm. We conducted the tensile tests in accordance with the national standard GB/T 228.1-2021. Figure 9c shows the specimens after they reached the point of fracture.

**Figure 10:**
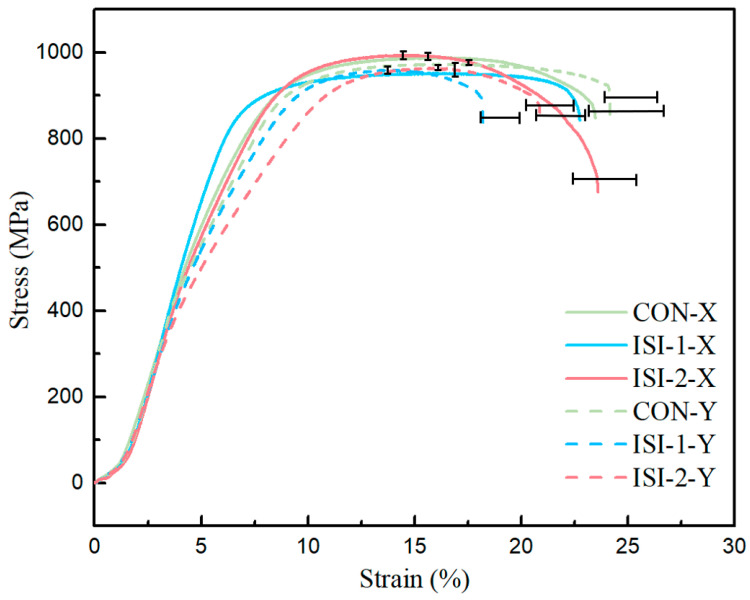
Tensile stress–strain curves in the X- and Y-directions under various scanning strategies.

Figure 10 presents a comparison of the tensile stress–strain curves in two directions under the three scanning strategies. It is observable that the tensile strength in the X-direction is higher under the Zone 2 scanning strategy than under the Zone 1 and continuous scanning strategies. This result occurs because the α phases that transform from the β matrix during the Zone 2 process possess relatively small dimensions and more grain boundaries. These boundaries effectively impede dislocation slip and consequently increase the tensile strength of the material. In contrast, the jump-scanning between sub-regions near the substrate allows the deposited areas to dissipate heat over a longer period. As a result, the cooling rate is higher than the rate observed during the continuous scanning process. This rapid solidification prevents gas bubbles from escaping from the molten pool in a timely manner. This phenomenon increases the porosity of the fabricated component and reduces its elongation. This trend is evident in the figure, where the elongation under the zone-scanning strategies is lower than the elongation under the continuous scanning strategy.

For tensile specimens that span multiple zones, particularly in the Y-direction, the elongation under the zone scanning strategy was significantly lower than the elongation observed under the continuous scanning strategy. This value was also lower than the elongation of specimens in the X-direction under the same strategy. Furthermore, the elongation of specimens under the Zone 1 scanning strategy was even lower. Microstructure has a significant impact on mechanical properties, including hardness, yield strength, and elongation. In this study, porosity and cracks are considered the key factors that influence mechanical properties. Based on the results for density and microstructure, zone scanning leads to a slight decrease in the density of the fabricated parts. However, the Zone 2 scanning strategy can improve the density because it varies the zone size between layers. During tensile tests, pores and cracks serve as the weakest initiation sites. The propagation of these defects accelerates the fracture process. The elongation of specimens under the Zone 1 scanning strategy is significantly lower than that of other specimens. This reduction is closely associated with the higher frequency of pores and cracks. Due to the perpendicular interlayer scanning vector used in the continuous scanning strategy, the tensile properties in the two directions show little difference. In this case, the elongation in the Y-direction is greater than the elongation in the X-direction. This phenomenon occurs because a reduction in the melt pool length during the laser metal deposition process can decrease Rayleigh instability and Marangoni forces. These changes may cause the melt to fail to overcome surface tension, which leads to inadequate wetting of the melt pool. During continuous scanning, heat dissipation is relatively slower during Y-direction scanning because of the substrate dimensions. This slower dissipation makes it easier to maintain a stable melt pool length. It also enhances the flow dynamics within the melt pool, which helps to reduce porosity and improve the elongation of the specimens.

**Figure 11:**
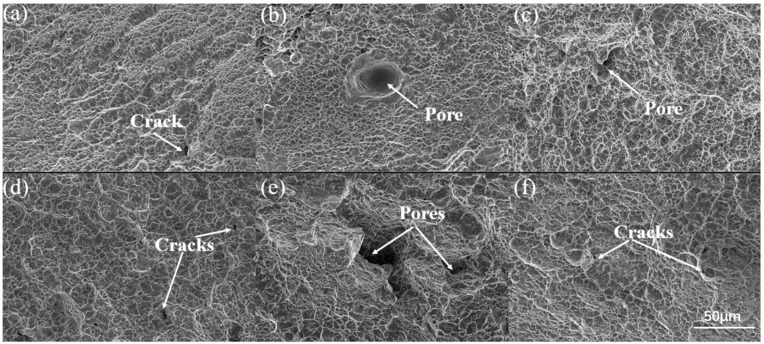
Tensile fracture morphologies of the specimens fabricated using different scanning strategies. (**a**,**d**) Continuous scanning, (**b**,**e**) Zone 1 scanning, and (**c**,**f**) Zone 2 scanning. The upper row (**a**–**c**) and lower row (**d**–**f**) correspond to the specimens tested along the X- and Y-directions, respectively. Representative cracks and pores are indicated by arrows. All SEM images were obtained at a magnification of 1000×, and the scale bar is 50 μm.

We examined the fracture surfaces of the tensile specimens in the X- and Y-directions across different scanning strategies to further elucidate the underlying fracture mechanisms. Figure 11 illustrates the fracture surface morphology of the specimens following the tensile tests. The fracture surfaces of all specimens exhibit a high density of dimples. This characteristic indicates that the specimens underwent a typical ductile fracture mode.

Furthermore, the figure shows that the fracture surfaces of the specimens produced by continuous scanning and Zone 2 scanning exhibit no significant voids. The distribution of dimples is uniform. Consequently, the tensile and yield strengths of these specimens exceed those of the specimens formed by Zone 1 scanning. Porosity has an adverse effect on tensile ductility. Therefore, the absence of voids on the fracture surface indicates that the material achieved a high relative density. For the Zone 1 specimens shown in Figure 11b,e, the formation of voids is likely due to unmelted powder particles. These particles can lead to the creation of gas pores during the laser metal deposition process. Additionally, lack-of-fusion defects occur at the boundaries between sub-regions. It can be inferred that the low elongation of the specimens formed using the Zone 1 scanning strategy is attributed to these gas pores and lack-of-fusion defects. Compared to the Zone 1 specimens, the specimens formed using the Zone 2 scanning strategy exhibit deeper and larger dimples. These features indicate sufficient plastic deformation and better elongation. The information reflected by the tensile fracture surface morphology aligns closely with the tensile property data.

## 4. Conclusions

This paper presents the results of fundamental process experiments for single-pass deposition. It discusses the effects of three key process parameters, including laser power, scanning speed, and powder feed rate, on the quality of the cladding layer. The study also optimizes the deposition process through an orthogonal experimental design. Furthermore, this work evaluates how three different scanning strategies affect the microstructure and mechanical properties of TC4 titanium alloy components. We fabricated large-scale block components using three specific methods. These methods include an orthogonal continuous scanning strategy, a sequential hopping scanning strategy with four rectangular partitions, and a proposed strategy that utilizes interlayer variable partition sizes. We compared the density, microstructural morphology, and hardness of the specimens produced under these scanning strategies. The main conclusions are listed below.

(1) Three key process parameters significantly influence the morphology and quality of single-pass cladding. Based on the evaluation of surface quality and deposition rate, the optimal parameters include a laser power of 2000 W, a scanning speed of 8 mm/s, and a powder feed rate of 4.44 g/min. Under these optimal conditions, the cladding height and width reach 1 mm and 4.16 mm, respectively.
(2) All three scanning strategies fabricated components with high relative density. The continuous scanning strategy resulted in a density of approximately 98.4%. In contrast, the four-zone scanning strategy yielded a lower density of about 93.6%. The interlayer variable partition size scanning strategy achieved a density of approximately 97.9%. This proposed strategy effectively mitigates the lack-of-fusion defects that typically arise at the boundaries of the four-rectangular partitions.
(3) The components fabricated via all three scanning strategies exhibit coarse primary β columnar grains that extend through multiple deposition layers. The bottom regions of these components consist of an acicular α’ phase. In the middle regions, a basket-weave microstructure forms because of heat accumulation. The top regions exhibit a shorter basket-weave microstructure due to the combined effects of heat accumulation and convective heat dissipation. The zone-scanning strategy facilitates effective heat dissipation by reducing the scan line length and implementing jump scanning between sub-regions. This approach results in a higher cooling rate. Consequently, the coarse growth of β grains is suppressed to a certain extent. Refined lamellar α grains are observable within the microstructure. Furthermore, the interlayer variable-partition-size scanning strategy yields the smallest grain sizes.
(4) The microhardness of the fabricated components across all scanning strategies exceeds that of the TC4 substrate. Furthermore, components produced using the interlayer variable partition size strategy exhibit the highest average hardness among the three methods.
(5) The mechanical properties of components fabricated with the interlayer variable partition size strategy are comparable to those produced by continuous scanning. Because of the presence of porosity, the ductility of all components produced via partitioned scanning is compromised. Consequently, their elongation is lower than the elongation of components produced by continuous scanning. Specimens from all three scanning strategies exhibit a typical ductile fracture mode.

## Figures and Tables

**Table 1:** Chemical composition of TC4 titanium alloy powder (wt.%).

Elments	Al	V	Fe	C	O	N	H	Ti
**content**	5.50–6.75	3.5–4.5	≤0.3	≤0.08	≤0.20	≤0.05	≤0.015	Bal.

**Table 2:** Orthogonal experimental design for process parameters.

No.	Laser Power (W)	Powder Feed Rate (g/min)	Scan Speed (mm/s)
1	1100	2.83	6
2	1100	3.64	8
3	1100	4.44	10
4	1100	5.25	12
5	1400	2.83	8
6	1400	3.64	6
7	1400	4.44	12
8	1400	5.25	10
9	1700	2.83	10
10	1700	3.64	12
11	1700	4.44	6
12	1700	5.25	8
13	2000	2.83	12
14	2000	3.64	10
15	2000	4.44	8
16	2000	5.25	6

**Table 3:** Geometric parameters of the selected cladding tracks.

Cladding Pass Number	Melting Point (mm)	Melt Width (mm)
5	0.51	3.18
6	0.90	3.47
9	0.62	3.61
10	0.62	3.34
12	1.05	3.69
13	0.62	3.28
14	0.69	4.10
15	1.01	4.16

## Data Availability

The original contributions presented in this study are included in the article. Further inquiries can be directed to the corresponding author.
